# The Effect of 2.45 GHz Radiofrequency Electromagnetic Radiation on Components of the Hypothalamic–Pituitary–Gonadal Axis in Male Rats

**DOI:** 10.3390/ijms27104582

**Published:** 2026-05-20

**Authors:** Sivasatyan Vijay, Siti Fatimah Ibrahim, Khairul Osman, Aini Farzana Zulkefli, Mohd Farisyam Mat Ros, Norazurashima Jamaludin, Syed Muhamad Asyraf Syed Taha, Atikah Hairulazam, Farah Hanan Fathihah Jaffar

**Affiliations:** 1 Department of Physiology, Faculty of Medicine, Universiti Kebangsaan Malaysia (UKM), Cheras, Kuala Lumpur 56000, Malaysiaainifarzana@hctm.ukm.edu.my (A.F.Z.); mdfarisyam@ukm.edu.my (M.F.M.R.);; 2 Forensic Science Program, Center for Diagnostics, Therapeutics, and Investigative Studies (CODTIS), Faculty of Health Sciences, Universiti Kebangsaan Malaysia (UKM), Jalan Raja Muda Abdul Aziz, Kuala Lumpur 50300, Malaysia; khairos@ukm.edu.my (K.O.); norazurashima@ilkkm.edu.my (N.J.)

**Keywords:** kisspeptin, GnRH, FSH, LH, RF-EMR, testosterone

## Abstract

The brain and testes are connected via the hypothalamic–pituitary–gonadal (HPG) axis. Both are vulnerable to radiofrequency electromagnetic radiation (RF-EMR). However, no comprehensive study had evaluated the effects of RF-EMR on key hormones along this axis. Hereby, this study evaluated the effect of RF-EMR on the hormonal changes along the axis, including the neuropeptide kisspeptin. A total of 18 (N = 18) adult Sprague–Dawley rats were divided into three groups (n = 6): Control, 4 h, and 24 h. The Control group was sham-exposed to an inactive router. The exposed groups were subjected to 2.45 GHz RF-EMR for 4 and 24 h daily, for 60 days at a 20 cm distance. The power density was 0.141 W/m^2^ with a whole-body specific absorption rate (SAR) of 0.41 W/kg. No significant changes were observed in hypothalamic *Kiss1* gene expression or serum kisspeptin levels. GnRH levels increased significantly in both exposed groups, while FSH and LH remained unchanged. Testicular testosterone was significantly reduced in the 24 h group, while serum testosterone was elevated in the 24 h group compared to the 4 h group. In conclusion, prolonged 2.45 GHz RF-EMR exposure caused selective changes in components of the HPG axis, particularly involving GnRH and testosterone, suggesting potential endocrine effects on male reproductive regulation.

## 1. Introduction

The impact of radiofrequency electromagnetic radiation (RF-EMR) from wireless devices on the male reproductive system has been the subject of numerous studies. RF-EMR emitted from wireless devices is one of the spectrums in the electromagnetic field (EMF) and classified as non-ionizing radiation. It is well known that non-ionizing radiation lacks the energy to break the chemical bond of a molecule [1]. Nevertheless, the fluid nature [2] and the high-water composition of the testes [3] consequently allow for the absorption of energy from the radiation, making it one of the most susceptible organs towards RF-EMR exposure [4,5].

One of the widely reported parameters that was affected by the RF-EMR exposure in animal studies is the testicular structure, which showed various degenerative changes [6,7,8]. In addition, there is also an increase in evidence that shows the testicular internal niche was in imbalance due to inflammation [9] and oxidative stress [6,10]. Consequently, the disturbance of the testicular microenvironment was reflected in compromised sperm quality, including reduced sperm concentration [11,12], motility and viability [13]. On the same note, it was also reported that there was an increase in sperm DNA damage [14,15] following the RF-EMR exposure.

Apart from the testes, the brain was another organ that was susceptible to RF-EMR exposure [16,17,18], partly due to its similar characteristics with the testes on fluidity [19,20]. Interconnected through the hypothalamus–pituitary–gonadal (HPG) axis, the brain regulates spermatogenesis in the testis through a strict regulation of the male reproductive hormones [21]. The hypothalamus initiates this regulatory process by the pulsatile release of the gonadotropin-releasing hormones (GnRH) [22]. GnRH secretion is, in turn, regulated by kisspeptin (Kp), a neuropeptide produced by *Kiss1*-expressing neurons [23] found in the hypothalamic region of the brain [24]. It is the modulation of Kp that determines the pulsatile release of GnRH [24,25].

Once released, the GnRH stimulates the anterior pituitary to secrete luteinizing hormone (LH) and follicle-stimulating hormone (FSH) [26]. LH exerts its effect on Leydig cells in the testes to induce secretion of testosterone, while FSH targets Sertoli cells to support proper spermatogenesis [27]. Eventually, testosterone exerts negative feedback on the hypothalamus and the anterior pituitary, which subsequently causes declining secretion of the Kp, GnRH, FSH and LH [28].

Though the outcome of the RF-EMR exposure highlighted the damaging effect on male reproductive organs and spermatogenesis outcome, the broader impact on hormonal changes along the HPG axis remains underexplored. As both the brain and the testes are susceptible organs to RF-EMR exposure, they are interlinked via hormonal communication, and any hormonal disruptions could significantly affect male reproductive outcomes.

Since hormonal balance is crucial to maintain spermatogenic function properly, this study aimed to evaluate the changes in key hormones of the HPG, including Kp, following exposure. It is imperative to understand the potential consequences of RF-EMR from Wi-Fi devices on male reproductive hormone holistically for developing strategies to mitigate these effects while maintaining continuous Wi-Fi exposure.

## 2. Results

### 2.1. The Expression Level of Kiss1 mRNA

**Figure 1:**
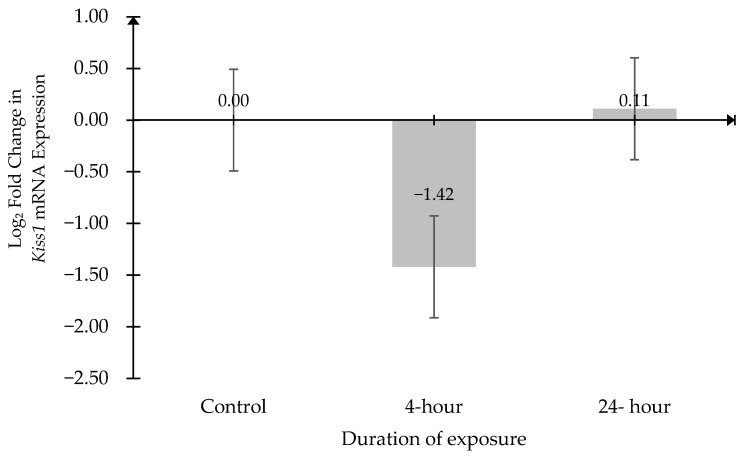
Log_2_ Fold Change in *Kiss1* mRNA expression across experimental groups. Data were expressed as relative fold change compared with the Control group (baseline = 0) and presented as mean ± SEM (n = 6). No significant difference between the groups.

*Kiss1* mRNA expression was normalized against the mRNA expression of *β-actin* and *glyceraldehyde-3-phosphate dehydrogenase* (*Gapdh*). Data were expressed as log2 fold change relative to the Control group (Figure 1). No significant differences in *Kiss1* mRNA expression among the groups. The comparison between the Control and 4 h groups yielded a 95% confidence interval of [−2.623, 5.470] (*p* = 0.6145). Similarly, no significant difference was observed between the Control and 24 h groups, with a 95% confidence interval of [−4.374, 4.157] (*p* = 0.9973).

### 2.2. The Kisspeptin Level in the Serum

**Figure 2:**
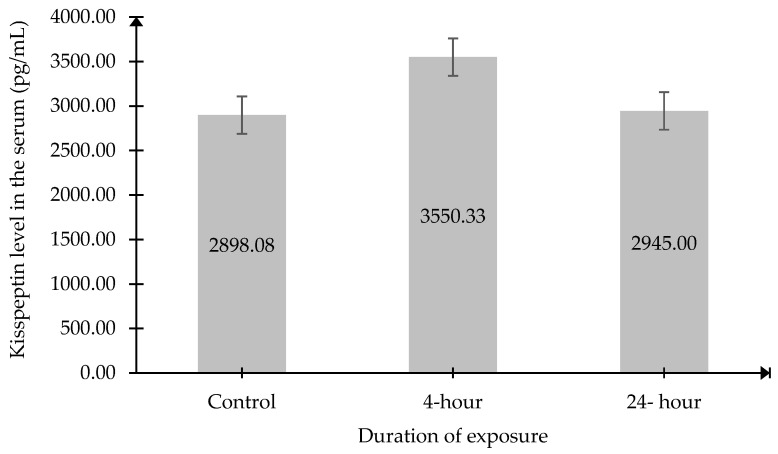
Serum kisspeptin levels in each experimental group. Data were presented as mean ± SEM (n = 6). No significant difference between the groups.

Serum kisspeptin levels did not show any significant differences among the groups exposed to different durations of 2.45 GHz RF-EMR (Figure 2).

### 2.3. Gonadotropin-Releasing Hormone (GnRH) Level in the Serum

**Figure 3:**
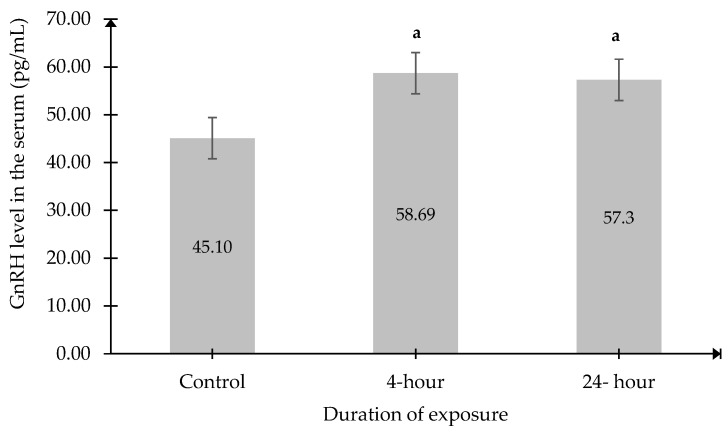
Serum GnRH levels in each experimental group. Data was presented as mean ± SEM (n = 6). ^a^ indicates a significant difference between the 4 h and 24 h groups compared with the Control group (*p* < 0.05). No significant difference between other groups.

Although no significant changes were observed in serum Kp levels, a significant increase in serum GnRH levels was recorded in the 4 h and 24 h exposed groups compared to the Control group (Figure 3).

### 2.4. Follicle-Stimulating Hormone (FSH) and Luteinizing Hormone (LH) Level in the Serum

**Figure 4:**
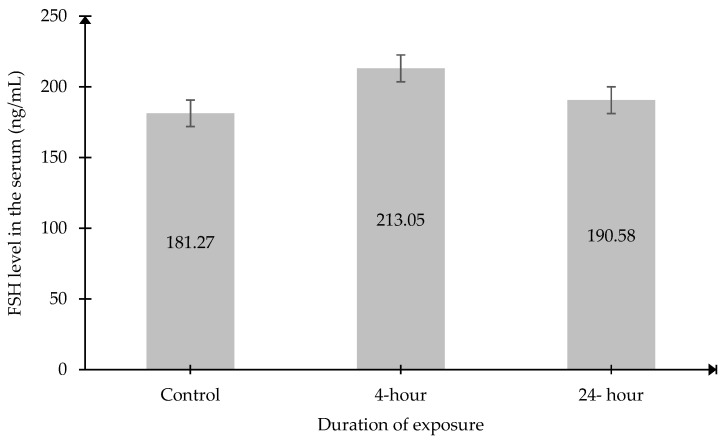
Serum FSH levels. Data was presented as mean ± SEM (n = 6). No significant differences between the group.

**Figure 5:**
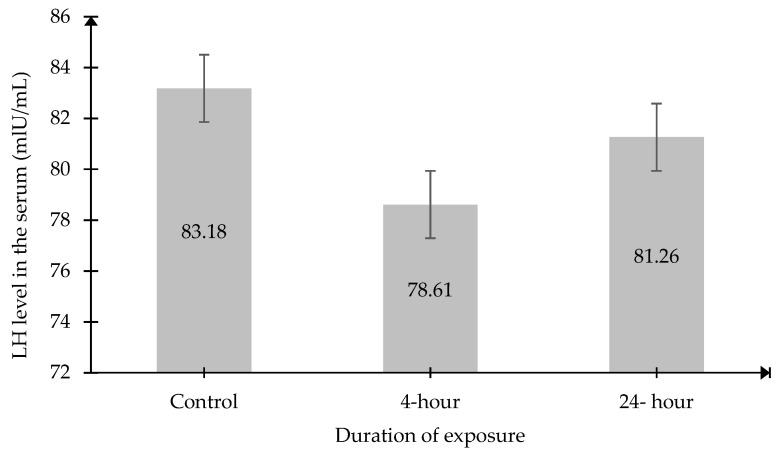
Serum LH levels. Data was presented as mean ± SEM (n = 6). No significant differences between the groups.

The results also demonstrated that there were no significant changes in serum FSH and LH levels in both exposed groups compared to the Control group (Figure 4 and Figure 5).

### 2.5. Testosterone Level in the Serum and Testicular Tissue

**Figure 6:**
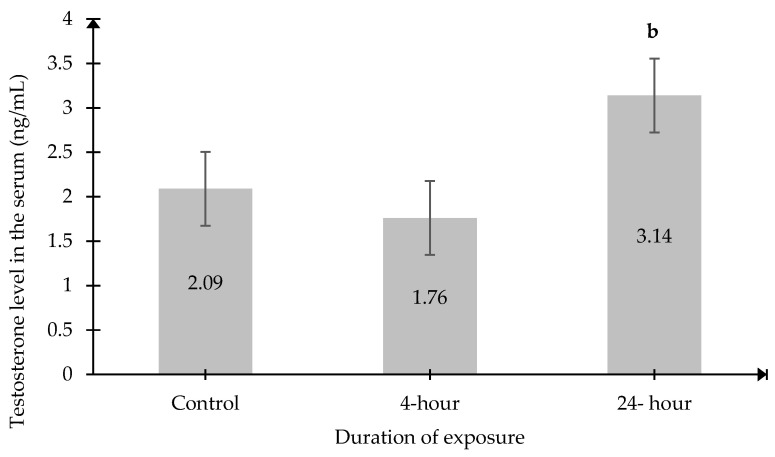
Serum testosterone levels. Data was presented as mean ± SEM (n = 6). ^b^ indicates a significant difference between the 24 h and 4 h groups (*p* < 0.05). No significant difference between other groups.

Significant changes in testosterone levels were recorded in both serum and testicular tissue. Serum testosterone levels in the 24 h exposed group (3.14 ng/mL ± 0.3996) were significantly higher than those in the 4 h exposed group (1.76 ng/mL ± 0.2955, *p* < 0.05) (Figure 6). However, no significant differences were observed between either the exposed group or the Control group.

**Figure 7:**
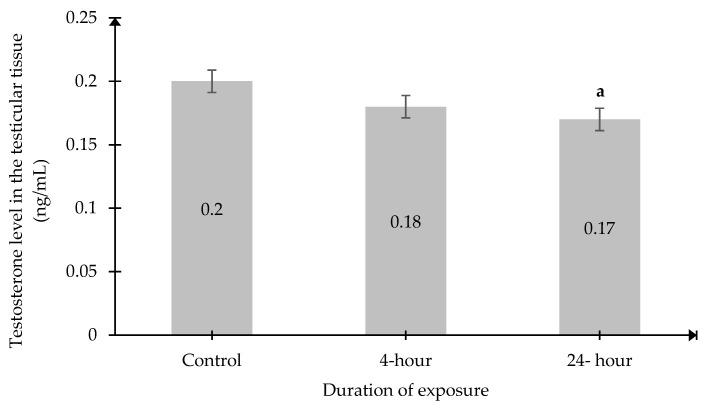
Testosterone levels in testicular tissue. Data was presented as mean± SEM (n = 6). ^a^ indicates a significant difference between the 24 h group and the Control group (*p* < 0.05). No significant difference between the other groups.

In contrast, intratesticular testosterone levels in the 24 h exposure group (0.1664 ± 0.003 units) were significantly lower than those in the Control group (0.1959 ± 0.0086 units, *p* < 0.05) (Figure 7).

## 3. Discussion

Testosterone [11,12,29,30], FSH and LH [31] are among the hormones previously investigated in several studies concerning the RF-EMR exposure from wireless devices. However, the effect of RF-EMR on the full spectrum of hormones along the hypothalamic–pituitary–gonadal (HPG) axis, including the key regulator kisspeptin (Kp), remains poorly understood. To the best of our knowledge, few studies have simultaneously evaluated multiple hormonal components across the HPG axis despite growing interest in RF-EMR exposure.

In this study, no significant changes were observed in *Kiss1* mRNA levels in the hypothalamic tissue of male rats exposed to 2.45 GHz RF-EMR. Consistently, no significant changes were also recorded in serum Kp levels following exposure. Notably, the corresponding wavelength at this frequency is approximately 12.2 cm [32], which is theoretically sufficient to penetrate the rat skull [33] and reach the underlying brain tissue where it could potentially influence *Kiss1* mRNA and protein expression. Yet, the extent to which this translates into biological effects on *Kiss1* expression remains uncertain.

However, as the exposure was conducted at 20 cm, the effective energy delivered to the brain may have been attenuated, potentially reducing the effective dose delivered to hypothalamic regions. The exposure device was positioned 20 cm from the animal cages, consistent with previous rodent studies [7,31,34] and aligned with international safety guidelines from the Federal Communications Commission (FCC) and International Commission on Non-Ionizing Radiation Protection [35,36], thereby ensuring far-field exposure conditions that approximate realistic user–device separation.

Additionally, the animals were freely moving within the cage, leading to variable orientation and inconsistent exposure of specific anatomical regions, particularly the head, thereby introducing exposure heterogeneity across animals. Nevertheless, this setup was applied to enable prolonged exposure, up to 24 h per day for two months, to imitate real-life exposure. Therefore, the lack of observable changes in hypothalamic *Kiss1* mRNA expression and serum Kp levels may be attributed to insufficient or inconsistent exposure of the target brain regions. Future studies may consider positioning the emitting device in closer proximity to the animal’s head with controlled movement restriction to achieve more localized and consistent RF-EMR exposure.

The lack of changes in serum Kp levels may also reflect the complex sources and circulation of Kp. Centrally produced Kp is largely confined to the hypophyseal portal circulation, with only a small fraction entering systemic circulation, and it generally cannot cross the blood–brain barrier (BBB) [37,38,39,40]. The small amount of Kp detected in serum may originate from peripheral tissues such as the liver, which also produces and secretes Kp into the blood [41]. This variability may potentially confound systemic measurements. To address these limitations, future studies could evaluate Kp levels directly in the hypophyseal portal blood, which would provide a more accurate assessment of its central regulation of GnRH secretion.

Regardless of the lack of changes in Kp levels, GnRH levels were significantly elevated in both exposed groups. This may suggest that exposure to 2.45 GHz RF-EMR influences GnRH secretion through mechanisms that are not solely dependent on Kp signaling. However, this suggestion remains hypothetical as Kp was measured only in serum despite its pulsatile release and confinement to the hypophyseal portal circulation. In this context, it is postulated that RF-EMR from 2.45 GHz exposure can trigger significant non-thermal effects on hypothalamic structures. One proposed non-thermal mechanism involves the production of reactive oxygen species (ROS) and a reduction in antioxidant defenses, which has been reported in previous studies to contribute to oxidative stress in neural tissues [42] and subsequent disruption of neuroendocrine homeostasis [30]. However, this was not directly measured in the present study.

The downstream effect of GnRH is to stimulate the anterior pituitary for the release of LH and FSH [21]. In the present study, no significant changes were recorded in the serum LH and FSH levels despite the significant increase in GnRH in both exposed groups. One possible explanation is that sustained elevation of GnRH may induce desensitization of GnRH receptors on pituitary cells [22,43]. Physiologically, GnRH must be secreted in a pulsatile manner to prevent receptor desensitization and to effectively stimulate gonadotroph cells to release LH and FSH [22]. The elevated GnRH observed in both exposed groups may therefore have exerted a tonic effect on pituitary gonadotrophs, resulting in unchanged LH and FSH levels.

LH secretion is also regulated by testosterone-mediated negative feedback [44]. Notably, serum testosterone levels were significantly higher in the 24 h group compared to the 4 h group. The increase in the testosterone level may have been sufficient to reduce LH pulse amplitude and prevent excessive Leydig cell stimulation [43], thereby contributing to the absence of significant LH changes in the 24 h group. Interestingly, testicular testosterone levels were significantly reduced in the 24 h group compared to controls, which may reflect a decline in Leydig cell steroidogenic capacity. Previous studies have also reported reduced testosterone levels in Leydig cells after being exposed to RF-EMR [29]. This impairment may be associated with changes in testicular histomorphometry following 2.45 GHz exposure. Supporting findings from a parallel study demonstrated edema formation between seminiferous tubules [8], potentially affecting Leydig cells located in the interstitial space. Additionally, oxidative stress resulting from an imbalance between ROS production and antioxidant capacity in testicular tissue [11,45,46] may contribute to disruption of testosterone production, although this was not directly assessed in the present study.

The discrepancies between serum and testicular testosterone suggest that the elevated serum testosterone after 24 h exposure does not necessarily indicate increased testicular biosynthesis. It may instead result from delayed metabolic clearance or disrupted hormone transport. RF-EMR exposure has been suggested to interfere with hepatic testosterone metabolism [47]. Testosterone is metabolized by cytochrome P450 enzymes in the liver into inactive metabolites such as androsterone and etiocholanolone [48]. RF-EMR has been associated with mitochondrial dysfunction and increased oxidative stress in hepatic and reproductive tissues [49,50], potentially impairing enzymatic activity and reducing testosterone clearance, thereby increasing its serum circulating levels. However, enzymatic or kinetic parameters were not evaluated in this study.

RF-EMR exposure may also influence serum testosterone through alterations in hormone-binding dynamics. In circulation, testosterone exists bound strongly to sex hormone-binding globulin (SHBG), loosely to albumin, or in a free biologically active form [46]. Reduced SHBG levels could potentially disrupt the balance between bound and free testosterone and impair hormonal clearance, contributing to prolonged elevation of serum testosterone [46].

Taken together, the present findings suggest that prolonged exposure to 2.45 GHz RF-EMR may influence selected components of the HPG axis through mechanisms partially independent of Kp signaling, possibly involving non-thermal modulation of hypothalamic GnRH regulation and downstream alterations in testosterone dynamics. However, these mechanisms remain hypothetical due to several limitations. In particular, Kp levels were measured only in serum rather than in the hypophyseal portal circulation, and oxidative stress markers were not assessed. Furthermore, enzymatic activity and kinetic parameters underlying hormonal metabolism were not evaluated, limiting mechanistic interpretation. The use of whole-body exposure with freely moving animals may have resulted in variable energy delivery to specific target tissues, particularly the brain. In addition, the relatively small sample size may limit the statistical power to detect subtle biological effects, and the absence of blinding may introduce potential bias despite the use of standardized procedures. Therefore, these findings should be interpreted with caution, and larger-scale studies incorporating localized exposure assessment, mechanistic biomarkers, and functional reproductive outcomes are warranted to validate and extend these observations. Despite these limitations, this study provides a comprehensive evaluation of multiple HPG axis components under realistic exposure conditions and adds important insight into the endocrine effects of chronic 2.45 GHz RF-EMR exposure.

## 4. Materials and Methods

### 4.1. Animals

A total of 18 (N = 18) adult male Sprague–Dawley rats of 8-week-old were obtained from the Laboratory Animal Resources Unit (LARU), Faculty of Medicine, Universiti Kebangsaan Malaysia (UKM). The initial body weight of the rats was approximately 250 ± 50 g. During the selection process, the animals were screened to ensure the absence of open wounds, abnormal growths, or deformities.

The rats were randomly and equally divided into three groups, with six rats (n = 6) in each group. In the absence of specific standardized guidelines for RF-EMR exposure in rodent models, the sample size was based on OECD Test Guideline 407, which recommends five males and five females. Nevertheless, six male rats per group were employed in accordance with institutional ethics committee approval and in alignment with the study’s targeted evaluation of male reproductive outcomes while adhering to the principle of reduction in animal use.

Each rat in each group was housed individually in a well-ventilated plastic cage (43 cm length × 16 cm wide × 29 cm height) without any movement restriction. The cages were placed in separate rooms within the animal house of the Faculty of Science and Technology, UKM. The animals were supplied with food pellets, clean tap water ad libitum and maintained in a controlled environment with an ambient temperature of 23 ± 5 °C and a 12:12 h light–dark cycle.

Throughout the experiment, daily animal monitoring was conducted to ensure the animals were not under any distress. Blinding was not feasible due to limited personnel resources. However, standardized procedures were applied consistently across all groups to minimize potential bias. Upon completion of the exposure, each animal was euthanized with a Ketamine–Tiletamine–Xylazine (KTX) cocktail intraperitoneally. All animal procedures were approved by the UKM Animal Ethical Committee (UKMAEC) with the approval reference number FP/2023/FARAH HANAN/15-FEB./1305-FEB.-2023-SEPT.-2024.

### 4.2. Exposure Setting

This study employed a TP-LINK AC570 Wireless Dual Band Wi-Fi Router Archer C20 (Shenzhen, China), which was equipped with three omnidirectional antennas. Among the three antennas, two generate a 2.45 GHz frequency, while one antenna generates a 5 GHz frequency. For this study, only the 2.45 GHz antennas were activated, whereas the single 5 GHz antenna was deactivated. According to the Maximum Permissible Exposure (MPE) report for this router, the power density of the antenna is 0.141 W/m^2^ for a 2.45 GHz frequency emission at a distance of 20 cm with a constant antenna gain of 3 dBi.

The three experimental groups in this study consist of the Control, 4 h, and 24 h groups. No movement restrictions were applied to the animals, as the exposure was conducted 24 h per day over 60 consecutive days. All the animal cages were positioned 20 cm away from the router. Through this setting, the animals in the exposed groups received a whole-body exposure with a SAR estimation of 0.41 W/kg.

The Control group served as sham exposed because the rats in this group were placed and maintained under the same conditions as the exposed groups, except that the router was turned off. In contrast, the 4 h and 24 h groups were exposed to an active Wi-Fi router for 4 h and 24 h daily, respectively. For the group that received Wi-Fi exposure, the Wi-Fi router was actively communicating with a Raspberry Pi computer (Cambridge University, Cambridge, UK) using a ping protocol via Bitvise SSH client version 8.18 software (Slovenia, Hungary, and USA). A total of 10 pings were sent each minute, and communication between the devices was done through the 802.11b/g/n standard.

To minimize the effects of external RF-EMR, aluminum foil was used to cover the walls of the exposure room, creating a Faraday cage effect. This setup also prevented animals in adjacent rooms from receiving signals from the exposure settings.

### 4.3. Serum, Testicular and Brain Tissue Sampling

Upon completion of the exposure, all the animals were euthanized, and the blood was drawn immediately via cardiac puncture. The blood was collected in BD Vacutainer SST II Advance Plus Blood Collection Tube (BD, Franklin Lakes, NJ, USA) and left undisturbed for at least 30 min at room temperature. Subsequently, the collection tubes were centrifuged at 1500× *g* for 10 min at 4 °C. The resulting serum was aliquoted and stored at −80 °C until analysis.

For testicular tissue sampling, both testes were meticulously dissected and cleaned of surrounding adipose tissue before being weighed and measured. One testis was preserved in 10% buffered neutral formalin (Merck, Darmstadt, Germany), while the other was snap-frozen in liquid nitrogen and stored at −80 °C for the subsequent hormonal analysis.

To collect the hypothalamus, the rat was further euthanized by decapitation by using a guillotine. The skull was carefully opened, and the brain was removed. There are two main populations of *Kiss1*-expressing cells, including the preoptic hypothalamic area (POA) and the mediobasal area of the hypothalamus (MBH). In rodents, POA is located within the rostral periventricular area of the third ventricle (RP3V), which includes the anteroventral periventricular nucleus (AVPV) and the adjacent preoptic periventricular nucleus. In contrast, the MBH population is found throughout the arcuate nucleus of the hypothalamus (ARH) [24]. The hypothalamus was dissected from the ventral region adjacent to the pituitary gland to ensure that all these regions were included. The harvested hypothalamus tissues were then immediately preserved in RNAlater solution, followed by a rapid snap-frozen procedure in liquid nitrogen. The hypothalamic samples were subsequently stored at −80 °C until further analysis [51,52].

### 4.4. Evaluation of Kisspeptin mRNA Expression

Frozen hypothalamic samples were thawed and weighed to obtain 20 mg of tissue. The hypothalamic tissue was homogenized in 350 μL of RNA Lysis Buffer Version 1 (RA) (Macherey-Nagel, Düren, Germany) supplemented with 3.5 μL of 2-mercaptoethanol using a syringe and needle [31]. Following this, the total RNA was extracted from the homogenized samples using the NucleoSpin RNA Kit (Macherey-Nagel, Düren, Germany) according to the manufacturer’s instructions. The extracted total RNA was eluted in 60 μL of RNase-free water (Macherey-Nagel, Düren, Germany). RNA concentration was measured using a NanoDrop DS-11+ spectrophotometer (DeNovix, Wilmington, DE, USA), and RNA concentration was reported in ng/μL [31].

The purity of the total RNA extract was assessed using the NanoDrop spectrophotometer based on the absorbance ratio at 260/280 nm (A260/280). RNA samples with A260/280 ratios ranging between 1.9 and 2.1 were considered suitable for quantitative polymerase chain reaction (qPCR) analysis.

Subsequently, the extracted RNA was reverse-transcribed into single-stranded complementary DNA (cDNA) using the High-Capacity cDNA Reverse Transcription Kit (Applied Biosystems, Carlsbad, CA, USA). Reverse transcription was performed by combining RNA with a master mix containing reverse transcription buffer, 100 mM deoxynucleotide triphosphates (dNTPs), random RT primers, and MultiScribe™ Reverse Transcriptase. The reaction conditions were as follows: 10 min at 25 °C, 120 min at 37 °C, and 5 min at 85 °C. The synthesized cDNA was stored at −20 °C until further use.

The qPCR analysis of the Kp mRNA expression was performed using PrecisionPLUS qPCR Master Mix (Primer Design, Manchester, UK). Each qPCR reaction consisted of 1 μL of cDNA template, 0.4 μL of primer, 5 μL of 2× qPCRBIO SyGreen Blue Mix Lo-ROX, and 3.6 μL of nuclease-free water (Primer Design, Manchester, UK). Reactions were performed in triplicate for each sample, with a final reaction volume of 10 μL. The *Kiss1* primers (Integrated DNA Technologies, Coralville, IA, USA) were used as the target gene, whereas *β-actin* and *Gapdh* primers (Integrated DNA Technologies, Coralville, IA, USA) were used as housekeeping genes. The detailed sequence of each primer is listed in Table 1.

The analysis was carried out using a CFX96™ Real-Time PCR System connected to a C1000 Thermal Cycler (Bio-Rad, Hercules, CA, USA) under the conditions stated in Table 2.

Cycle threshold (Ct) values obtained for each sample were used to determine mRNA expression levels using the relative quantification and comparative Ct method. Ct values of the target gene were normalized against housekeeping genes and compared with the Control group. Validation of the *Kiss1* qPCR product was performed using agarose gel electrophoresis to verify the expected amplicon size. This step was undertaken to ensure that the primers produced a single band of the correct size, thereby confirming the specificity and effectiveness of the amplification reaction.

### 4.5. Determination of Serum Kisspeptin, GnRH, FSH and LH

Frozen serum was thawed at room temperature to determine the serum levels of Kp, GnRH, FSH, LH, and testosterone by using enzyme-linked immunosorbent assay (ELISA). ELISA kits for Kp, FSH and LH (Wuhan, China) were based on the sandwich-ELISA method. The ELISA microplates were pre-coated with specific antibodies against Kp, FSH and LH.

A total of 100 μL serum samples and standard solutions were added in duplicate into separate wells of the microplate and incubated at 37 °C for 90 min. This was followed by the addition of 100 μL of biotinylated detection antibody and incubation for 1 h. Subsequently, 100 μL of horseradish peroxidase (HRP) conjugate solution was added and further incubated for 30 min and followed by five washing steps.

Subsequently, a total of 90 μL of substrate reagent was added and incubated for another 15 min until a blue color developed. Stop solution was then added, and the optical density (OD) was measured at 450 nm using a SpectraMax Plus 384 Microplate Reader (Molecular Devices, LLC, Downingtown, PA, USA). The OD values were directly proportional to the concentrations of FSH, LH, and kisspeptin. Standard curves for kisspeptin, FSH and LH were generated using a four-parameter logistic (4PL) regression model. An acceptable correlation coefficient of r ≥ 0.98 (R^2^ ≥ 0.98) was considered indicative of good assay performance. To eliminate inter-assay variability, all samples were analyzed within a single batch. The intra-assay coefficient of variation (CV) for these kits was maintained below 10%. The limits of detection (LOD) were 46.88 pg/mL for Kp, 1.88 ng/mL for FSH, and 0.94 mIU/mL for LH.

Determination of GnRH and testosterone levels was performed using ELISA kits based on the competitive ELISA method. Serum samples were used for the determination of GnRH levels, while both serum and testicular homogenate samples were used for testosterone analysis.

A total of 50 μL serum samples or testicular homogenate was added in duplicate into separate microplate wells. About 100 μL of biotinylated antibody was added immediately to each well. The microplate was incubated for 45 min at 37 °C. After one washing step, 100 μL of HRP-conjugated solution was added and incubated for an additional 30 min.

Following five washing steps, 90 μL of substrate reagent was added and incubated for 15 min until a blue color developed. Subsequently, 50 μL of stop solution was added. The optical density (OD) was measured at 450 nm using a SpectraMax Plus 384 Microplate Reader (Molecular Devices, LLC, Downingtown, PA, USA). The OD values were inversely proportional to the concentrations of testosterone and GnRH. Standard curves for GnRH and testosterone were generated using a four-parameter logistic (4PL) regression model as well, and an acceptable correlation coefficient of r ≥ 0.98 (R^2^ ≥ 0.98) was considered indicative of good assay performance.

Consistent with the sandwich assays, the competitive ELISAs were executed in a single batch to prevent batch-to-batch variation. The assays demonstrated an intra-assay CV of less than 10%, with LODs established at 9.38 pg/mL for GnRH and 0.17 ng/mL for testosterone.

### 4.6. Statistical Analysis

Statistical analysis for this study was performed using GraphPad Prism version 10 (GraphPad Software Inc., San Diego, CA, USA). Prior to the analysis of variance (ANOVA), the data normality and homogeneity across the group were verified using the Shapiro–Wilk test and the Brown–Forsythe test. One-way Analysis of Variance (ANOVA) with post hoc Tukey test was used to determine the effects of 2.45 GHz Wi-Fi exposure on kisspeptin levels, GnRH, FSH, LH, and testosterone levels. A *p*-value of ≤0.05 was considered statistically significant for all analyses.

## 5. Conclusions

This study shows that exposure to 2.45 GHz RF-EMR may influence certain hormone in the HPG axis, even in the absence of overt changes in the Kp pathway. Although serum Kp levels and hypothalamic *Kiss1* mRNA expression were unchanged, GnRH levels increased significantly. This may suggest that hypothalamic activity may be altered through kisspeptin-independent mechanisms. However, this remains hypothetical and needs further validation in future studies. The lack of corresponding changes in LH and FSH reflects the tightly regulated and compensatory nature of the HPG axis. Notably, most endocrine effects appeared time-independent, except for testicular testosterone, which showed time-dependent changes, pointing to possible progressive local disruption. Together, the observed effects of hormonal changes are not widespread across the axis but may indicate potential endocrine responses to RF-EMR exposure. However, the underlying mechanisms remain unclear and were not directly assessed in this study. Therefore, these findings should be interpreted with caution, and further studies incorporating functional reproductive outcomes and mechanistic investigations are warranted to better understand the biological significance of these effects.

## Figures and Tables

**Table 1:** Primer sequence for the target and housekeeping gene.

Gene	Primer Sequence (5′–3′)		NCBI Accession Number	Product Size (bp)
*Kiss1*	Forward	CTGCTGCTTCTCCTCTGTGTG	NM_181692	117
	Reverse	CCAGGCATTAACGAGTTCCT		
*Gapdh*	Forward	TCTCTGCTCCTCCCTGTTC	NM_017008 XM_216453	120
	Reverse	GTAACCAGGCGTCCGATAC		
*β-actin*	Forward	TCACTATCGGCAATGAGCG	NM_031144	143
	Reverse	GGCATAGAGGTCTTTACGGATG		

Source: Oligonucleotide specification sheet, Integrated DNA Technologies, Inc. (https://www.idtdna.com).

**Table 2:** qPCR Cycling Protocol.

Step	Temperature	Duration	Cycle
Enzyme activation	95 °C	2 min	-
Denaturation		10 s	40
Annealing	60 °C	60 s	
Melt Curve	65–95 °C	5 s	-

Source: Life Technologies Corporation (2011) [53].

## Data Availability

The original contributions presented in this study are included in the article. Further inquiries can be directed to the corresponding authors.

## Abbreviations

The following abbreviations are used in this manuscript:

4PL	Four Parameter Logistic
ARH	Arcuate Nucleus of the Hypothalamus
AVPV	Anteroventral Periventricular Nucleus
BBB	Blood–Brain Barrier
cDNA	Complementary Deoxyribonucleic Acid
cm	Centimeter
CT	Cycle Threshold
DNA	Deoxyribonucleic Acid
dNTPs	Deoxynucleoside Triphosphates
FSH	Follicle-Stimulating Hormone
g	Gram
*Gapdh*	Glyceraldehyde 3-Phosphate Dehydrogenase
GHz	Gigahertz
GnRH	Gonadotropin-Releasing Hormone
HPG	Hypothalamic–Pituitary–Gonadal
Kp	Kisspeptin
KTX	Ketamine–Xylazine
LH	Luteinizing Hormone
MBH	Medial Basal Hypothalamus
MPE	Maximum Permissible Exposure
mRNA	Messenger Ribonucleic Acid
OD	Optical Density
POA	Preoptic Area
qPCR	Quantitative Polymerase Chain Reaction
RF-EMR	Radiofrequency Electromagnetic Radiation
SAR	Specific Absorption Rate
SHBG	Sex Hormone-Binding Globulin
Wi-Fi	Wireless Fidelity

## References

[1] Omer H. (2021). Radiobiological Effects and Medical Applications of Non-Ionizing Radiation. Saudi J. Biol. Sci..

[2] Karaman I.P., Coskun O., Senol N., Sahin M., Comlekci S. (2024). Alleviative Effect of Quercetin on Rat Testicular against 2600 MHz Electromagnetic Field. Int. J. Radiat. Res..

[3] Reinoso R.F., Telfer B.A., Rowland M. (1997). Tissue water content in rats measured by desiccation. J. Pharmacol. Toxicol. Methods.

[4] Mahmoudi R., Karbalay-Doust S., Masoudi E., Jafari-Barmak M., Ghanbri A., Nikseresht M., Mohammad Javad Mortazavi S., Alireza Mortazavi S. (2025). Mitigating Heat-Induced Sperm Damage and Testicular Tissue Abnormalities: The Protective Role of Radiofrequency Radiation from Wi-Fi Routers in Rodent Models. J. Biomed. Phys. Eng..

[5] Kaur P., Rai U., Singh R. (2023). Genotoxic Risks to Male Reproductive Health from Radiofrequency Radiation. Cells.

[6] Jaffar F.H.F., Osman K., Hui C.K., Zulkefli A.F., Ibrahim S.F. (2024). Effect of Wi-Fi Exposure and Edible Bird Nest Supplementation on the Testicular Oxidative Stress Status and Sperm Quality in Male Sprague-Dawley Rat Pups. Int. J. Radiat. Res..

[7] Shokri S., Soltani A., Kazemi M., Sardari D., Mofrad F.B. (2015). Effects of Wi-Fi (2.45 GHz) Exposure on Apoptosis, Sperm Parameters and Testicular Histomorphometry in Rats: A Time Course Study. Cell J..

[8] Vijay S., Ibrahim S.F., Osman K., Zulkefli A.F., Mat Ros M.F., Jamaludin N., Syed Taha S.M.A., Hairulazam A., Jaffar F.H.F. (2025). Histomorphometry and Sperm Quality in Male Rats Exposed to 2.45 GHz Wi-Fi. Reproduction.

[9] Ok F., Emre M., Bisgin A., Jafarguliyev S., Boga I., Cetiner S., Yesyet G., Bagir E., Bayazit Y., Doran S. (2024). Effect of 900 MHz Radiofrequency Electromagnetic Radiation Emitted from Mobile Phone on Testicular Immunity and the Associated Risk of Testicular Germ Cell Tumor. Tumor Discov..

[10] Gupta V., Srivastava R. (2025). Amelioration and Immuno-Modulation by Ashwagandha on Wi-Fi Induced Oxidative Stress in Regulating Reproduction Via Estrogen Receptor Alpha in Male Japanese Quail. Reprod. Sci..

[11] Jamaludin N., Ibrahim S.F., Jaffar F.H.F., Zulkefli A.F., Osman K. (2025). The Influence of 2.45 GHz Wi-Fi Exposure Duration on Sperm Quality and Testicular Histopathology: An Exploration of Peroxidative Injury. Antioxidants.

[12] Maluin S.M., Jaffar F.H.F., Osman K., Zulkefli A.F., Mat Ros M.F., Ibrahim S.F. (2024). Exploring Edible Bird Nest’s Potential in Mitigating Wi-Fi’s Impact on Male Reproductive Health. Reprod. Med. Biol..

[13] Chu K.Y., Khodamoradi K., Blachman-Braun R., Dullea A., Bidhan J., Campbell K., Zizzo J., Israeli J., Kim M., Petrella F. (2023). Effect of Radiofrequency Electromagnetic Radiation Emitted by Modern Cellphones on Sperm Motility and Viability: An In Vitro Study. Eur. Urol. Focus.

[14] Koohestanidehaghi Y., Khalili M.A., Dehghanpour F., Seify M. (2024). Detrimental Impact of Cell Phone Radiation on Sperm DNA Integrity. Clin. Exp. Reprod. Med..

[15] Zahmatkesh P., Mohammadi A., Mashhadi R., Khatami F., Mirzaei A., Zareian Baghdadabad L., Khalili F., Gholami K., Rahimnia R., Noori N. (2024). The Impact of Radiofrequency Electromagnetic Waves on DNA Fragmentation Index and Spermatogenesis-Related Genes Expression in Rats. Int. J. Med. Toxicol. Forensic Med..

[16] Bektas H., Algul S., Altindag F., Yegin K., Akdag M.Z., Dasdag S. (2022). Effects of 3.5 GHz Radiofrequency Radiation on Ghrelin, Nesfatin-1, and Irisin Level in Diabetic and Healthy Brains. J. Chem. Neuroanat..

[17] Haifa O., Mariem T., Mohsen S., Hafedh A., Mohamed A. (2021). Behavioral Impairments and Biochemical Alterations in Brain Following Exposure to WiFi Radiation and Aluminum in Rats. Int. J. Radiol. Radiat. Oncol..

[18] Osama Mohamed A., Mohamed Naguib Abdel-Hafez S., Ahmed Ibrahim R., Ahmed Refaai R. (2022). The Possible Effects of Electromagnetic Waves of Wi-Fi Router on the Hippocampus of Male Rats. Minia J. Med. Res..

[19] Li M., Shi Q., Jiang X., Liu X., Han W., Fan X., Li P., Qi K. (2022). Paternal Preconceptional Diet Enriched with N-3 Polyunsaturated Fatty Acids Affects Offspring Brain Function in Mice. Front. Nutr..

[20] Ye X., Li Y., González-Lamuño D., Pei Z., Moser A.B., Smith K.D., Watkins P.A. (2024). Role of ACSBG1 in Brain Lipid Metabolism and X-Linked Adrenoleukodystrophy Pathogenesis: Insights from a Knockout Mouse Model. Cells.

[21] Li L., Lin W., Wang Z., Huang R., Xia H., Li Z., Deng J., Ye T., Huang Y., Yang Y. (2024). Hormone Regulation in Testicular Development and Function. Int. J. Mol. Sci..

[22] Marques P., De Sousa Lages A., Skorupskaite K., Rozario K.S., Anderson R.A., George J.T. (2024). Physiology of GnRH and Gonadotropin Secretion. Endotext [Internet].

[23] Piet R., de Croft S., Liu X., Herbison A.E. (2015). Electrical Properties of Kisspeptin Neurons and Their Regulation of GnRH Neurons. Front. Neuroendocrinol..

[24] Delli V., Silva M.S.B., Prévot V., Chachlaki K. (2021). The KiNG of Reproduction: Kisspeptin/ NNOS Interactions Shaping Hypothalamic GnRH Release. Mol. Cell. Endocrinol..

[25] Anderson R.A., Millar R.P. (2022). The Roles of Kisspeptin and Neurokinin B in GnRH Pulse Generation in Humans, and Their Potential Clinical Application. J. Neuroendocrinol..

[26] Xie Q., Kang Y., Zhang C., Xie Y., Wang C., Liu J., Yu C., Zhao H., Huang D. (2022). The Role of Kisspeptin in the Control of the Hypothalamic-Pituitary-Gonadal Axis and Reproduction. Front. Endocrinol..

[27] Peper J.S., Brouwer R.M., van Leeuwen M., Schnack H.G., Boomsma D.I., Kahn R.S., Hulshoff Pol H.E. (2010). HPG-Axis Hormones during Puberty: A Study on the Association with Hypothalamic and Pituitary Volumes. Psychoneuroendocrinology.

[28] Corradi P.F., Corradi R.B., Greene L.W. (2016). Physiology of the Hypothalamic Pituitary Gonadal Axis in the Male. Urol. Clin. N. Am..

[29] Lin Y.-Y., Wu T., Liu J.-Y., Gao P., Li K.-C., Guo Q.-Y., Yuan M., Lang H.-Y., Zeng L.-H., Guo G.-Z. (2017). 1950MHz Radio Frequency Electromagnetic Radiation Inhibits Testosterone Secretion of Mouse Leydig Cells. Int. J. Environ. Res. Public Health.

[30] Maluin S.M., Osman K., Jaffar F.H.F., Ibrahim S.F. (2021). Effect of Radiation Emitted by Wireless Devices on Male Reproductive Hormones: A Systematic Review. Front. Physiol..

[31] Jaffar F.H.F., Osman K., Hui C.K., Zulkefli A.F., Ibrahim S.F. (2022). Long-Term Wi-Fi Exposure From Pre-Pubertal to Adult Age on the Spermatogonia Proliferation and Protective Effects of Edible Bird’s Nest Supplementation. Front. Physiol..

[32] Kesari K.K., Agarwal A., Henkel R. (2018). Radiations and Male Fertility. Reprod. Biol. Endocrinol..

[33] O’Reilly M.A., Muller A., Hynynen K. (2011). Ultrasound Insertion Loss of Rat Parietal Bone Appears to Be Proportional to Animal Mass at Submegahertz Frequencies. Ultrasound Med. Biol..

[34] Oh J.J., Byun S.S., Lee S.E., Choe G., Hong S.K. (2018). Effect of Electromagnetic Waves from Mobile Phones on Spermatogenesis in the Era of 4G-LTE. BioMed Res. Int..

[35] Federal Communications Commission (2020). Human Exposure to Radiofrequency Electromagnetic Fields and Reassessment of FCC Radiofrequency Exposure Limits and Policies. https://www.federalregister.gov/documents/2020/04/01/2020-02745/human-exposure-to-radiofrequency-electromagnetic-fields-and-reassessment-of-fcc-radiofrequency.

[36] Ziegelberger G., Croft R., Feychting M., Green A.C., Hirata A., d’Inzeo G., Jokela K., Loughran S., Marino C., Miller S. (2020). Guidelines for Limiting Exposure to Electromagnetic Fields (100 KHz to 300 GHz). Health Phys..

[37] Comninos A.N., Wall M.B., Demetriou L., Shah A.J., Clarke S.A., Narayanaswamy S., Nesbitt A., Izzi-Engbeaya C., Prague J.K., Abbara A. (2017). Kisspeptin Modulates Sexual and Emotional Brain Processing in Humans. J. Clin. Investig..

[38] Luedde M., Spehlmann M.E., Hippe H.J., Loosen S.H., Roy S., Cardenas D.V., Vucur M., Frey N., Koch A., Luedde T. (2018). Serum Levels of Kisspeptin Are Elevated in Critically Ill Patients. PLoS ONE.

[39] Skorupskaite K., George J.T., Anderson R.A. (2014). The Kisspeptin-GnRH Pathway in Human Reproductive Health and Disease. Hum. Reprod. Update.

[40] Yoshihisa U., Vutha P., Hiroko T., Kei-ichiro M. (2016). The Roles of Kisspeptin Revisited: Inside and Outside the Hypothalamus. J. Reprod. Dev..

[41] Meccariello R., Fasano S., Pierantoni R. (2020). Kisspeptins, New Local Modulators of Male Reproduction: A Comparative Overview. Gen. Comp. Endocrinol..

[42] Roychoudhury S., Chakraborty S., Choudhury A.P., Das A., Jha N.K., Slama P., Nath M., Massanyi P., Ruokolainen J., Kesari K.K. (2021). Environmental Factors-Induced Oxidative Stress: Hormonal and Molecular Pathway Disruptions in Hypogonadism and Erectile Dysfunction. Antioxidants.

[43] O’Donnell L., Stanton P., de Kretser D.M. (2017). Endocrinology of the Male Reproductive System and Spermatogenesis. Endotext [Internet].

[44] Ilie I.R. (2020). Introduction to Endocrinology.

[45] Jangid P., Rai U., Singh R. (2024). Radio Frequency Electromagnetic Radiations Interfere with the Leydig Cell Functions In-Vitro. PLoS ONE.

[46] Nassar G.N., Leslie S.W. (2023). Testosterone. Physiology, Testosterone.

[47] Borzoueisileh S., Monfared A.S., Ghorbani H., Mortazavi S.M.J., Zabihi E., Pouramir M., Doustimotlagh A.H., Shafiee M., Niksirat F. (2020). Assessment of Function, Histopathological Changes, and Oxidative Stress in Liver Tissue Due to Ionizing and Non-Ionizing Radiations. Casp. J. Intern. Med..

[48] Schiffer L., Barnard L., Baranowski E.S., Gilligan L.C., Taylor A.E., Arlt W., Shackleton C.H.L., Storbeck K.-H. (2019). Human Steroid Biosynthesis, Metabolism and Excretion Are Differentially Reflected by Serum and Urine Steroid Metabolomes: A Comprehensive Review. J. Steroid Biochem. Mol. Biol..

[49] Santini S.J., Cordone V., Falone S., Mijit M., Tatone C., Amicarelli F., Di Emidio G. (2018). Role of Mitochondria in the Oxidative Stress Induced by Electromagnetic Fields: Focus on Reproductive Systems. Oxid. Med. Cell. Longev..

[50] Schuermann D., Mevissen M. (2021). Manmade Electromagnetic Fields and Oxidative Stress—Biological Effects and Consequences for Health. Int. J. Mol. Sci..

[51] Góźdź A., Szczepańska-Sadowska E., Maśliński W., Kumosa M., Szczepańska K., Dobruch J. (2003). Differential Expression of Vasopressin V1a and V1b Receptors MRNA in the Brain of Renin Transgenic TGR(MRen2)27 and Sprague–Dawley Rats. Brain Res. Bull..

[52] Mosili P., Mkhize B.C., Ngubane P., Sibiya N., Khathi A. (2020). The Dysregulation of the Hypothalamic–Pituitary–Adrenal Axis in Diet-Induced Prediabetic Male Sprague Dawley Rats. Nutr. Metab..

[53] Life Technologies Corporation (2011). SYBR^®^ Green PCR Master Mix and SYBR^®^ Green RT-PCR Reagents Kit User Guide.

